# Distribution of Fitness in Populations of Dengue Viruses

**DOI:** 10.1371/journal.pone.0107264

**Published:** 2014-09-15

**Authors:** Md Abu Choudhury, William B. Lott, John Aaskov

**Affiliations:** 1 Institute of Health and Biomedical Innovation, Queensland University of Technology, Brisbane, Queensland, Australia; 2 School of Chemistry, Physics, and Mechanical Engineering, Science and Engineering Faculty, Queensland University of Technology, Brisbane, Queensland, Australia; University of California Davis, United States of America

## Abstract

Genetically diverse RNA viruses like dengue viruses (DENVs) segregate into multiple, genetically distinct, lineages that temporally arise and disappear on a regular basis. Lineage turnover may occur through multiple processes such as, stochastic or due to variations in fitness. To determine the variation of fitness, we measured the distribution of fitness within DENV populations and correlated it with lineage extinction and replacement. The fitness of most members within a population proved lower than the aggregate fitness of populations from which they were drawn, but lineage replacement events were not associated with changes in the distribution of fitness. These data provide insights into variations in fitness of DENV populations, extending our understanding of the complexity between members of individual populations.

## Introduction

Dengue viruses (DENVs) are the world’s most important mosquito-borne viral pathogens for humans in terms of morbidity, mortality and economic impact. DENVs consist of four antigenically distinct serotypes (DENV 1–4), which cause a wide spectrum of clinical manifestations. An estimated 3.6 billion people in the global population and approximately 120 million travellers are at risk of DENV infection. The number of dengue cases reported annually is 50–100 million, with approximately 24,000 deaths in children [1]–[4]. There is no commercially available DENV vaccine or DENV specific antiviral therapies, despite more than fifty years of research in this field [5].

DENV is a single stranded, positive sense RNA virus belonging to the genus *Flavivirus* (family *Flaviviridae*). Due to the error prone nature of the RNA-dependent RNA polymerase (RdRP) and genome recombination [6]–[8], DENVs raise significant genetic diversity during their replication [9]. Phylogenetic studies of DENV serotypes showed that they form diverse phylogenetic clusters, that consist of multiple distinct lineages [10], [11]. The lineage extinction and replacement on a regular basis is the most surprising feature of DENVs evolutionary dynamics [10]. A lineage that persists for a number of years at a given geographical location sometimes becomes extinct, as an entirely new lineage takes over [12]. Lineage replacement events on a regional scale are well documented. For example, DENV-1 lineage replacements were observed in Myanmar, Cambodia, Thailand in the late 1990s [13], in the mid-1990s [12], [14] and in the early 2000s [15] respectively. Similarly, DENV-2 lineage replacements were observed in Vietnam in the early 2000s [16]. DENV-3 lineage replacements were observed in Sri Lanka in the late 1980s [17], and in Thailand in the early 1990s [18]. And DENV-4 lineage replacements were observed in Puerto Rico during the 1980s and 1990s [19]. A more global lineage replacement event has also been reported [20], in which DENV-2 lineages from Southeast Asia displaced the American DENV-2 lineage in the Americas during the early 1990s.

Exploring the causes of DENV lineage replacement has important implications for dengue epidemiology and control [14], [17], [20]–[23]. As DENV antigenic properties often differ between lineages, understanding the mechanisms that underlie lineage turnover will influence vaccine design [14], [24], and the putative mechanisms of lineage replacement are used to develop prediction models for future dengue epidemics [14], [16]. Despite this potential significance, there are multiple explanations exists on whether lineage replacement events result from the random sampling of viral variants during genetic bottlenecks due to the stochastic nature of DENV transmission, or from variations in fitness within discrete viral populations. For the purposes of this work, fitness is defined as the ability of DENV-1 virions to replicate in cultured cells.

Some phylogenetic studies have suggested that observed DENV lineage replacement events were due to either a higher viraemia in the human host [16] or enhanced infectivity in mosquito vectors [14], [25]–[27], while others suggested that the data were more consistent with stochastic events [13], [18]. With respect to viral fitness in other systems, fewer than 5% of members of Vesicular stomatitis virus (VSV) populations were reported to be more fit than the population from which they were drawn [28], and mixtures of Ross river virus (RRV) populations containing less than 1% of a virulent strain nonetheless displayed a virulent phenotype [29]. Despite these observations, the distribution of fitness in DENV populations has been not yet been quantified and correlated with epidemiological patterns, like lineage extinction and replacement during transmission. Here, we measured distribution of fitness within DENV populations with differing epidemiological histories and correlated them with observed lineage extinction and replacement events.

## Materials and Methods

### Ethics statement

This study was approved by the Queensland University of Technology Research Ethics Unit (Ethics No. 0700000910). As no patient tissue was employed in this study, the University Ethics Unit did not require informed patient consent. All patient identifiers were removed from dengue virus samples before they were used for research purposes.

### Study population

Viruses were recovered from acute phase sera from dengue patients admitted to the Yangon Children’s Hospital [30]. Strains of DENV used in the study, which are described in Table 1, were passaged once in C6/36 before sequencing.

**Table 1:** Strains of DENV-1 used.

Serotype	Strain	Country	Date ofIsolation	Accessionnumber	Source	Passagenumber
DENV-1	31459	Myanmar	1998	AY588272	[67]	P1 in C6/36
	31987	Myanmar	1998	AY588273	[67]	P1 in C6/36
	32514	Myanmar	1998	AY600860	[67]	P1 in C6/36
	36957	Myanmar	2000	AY620951	[67]	P1 in C6/36
	43826	Myanmar	2001	DQ264966	[34]	P1 in C6/36
	44988	Myanmar	2002	AY726552	Unpublished	P1 in C6/36
	47317	Myanmar	2002	KF559253	This study	P1 in C6/36
	47662	Myanmar	2002	DQ265041	[34]	P1 in C6/36
	49440	Myanmar	2002	DQ265137	[34]	P1 in C6/36
	62690	Myanmar	2005	KF559255	This study	P1 in C6/36
	68417	Myanmar	2007	KF559256	This study	P1 in C6/36
	80579	Myanmar	2009	KF559257	This study	P1 in C6/36
	Infectious clone	Myanmar	2002	KF559254	This study	1 X BHK, 1 X C6/36
DENV-2	New Guinea C	New Guinea	1944	AF038403	[68]	Multiple, unknown
DENV-3	H87	Philippines	1956	M93130	[69]	Multiple, unknown
DENV-4	H241	Philippines	1956	AY947539	Novartis Institute forTropical Diseases	Multiple, unknown

### Cell lines and virus isolation

Cell lines were maintained in RPMI-1640 medium (Invitrogen) supplemented with 10% (v/v) heat-inactivated fetal bovine serum (FBS) (Invitrogen) and 1% (v/v) L-Glutamine (200 mM) Penicillin (10,000 units) Streptomycin (10 mg/ml) (Sigma). C6/36 cells were incubated at 30°C and all others were at 37°C in an atmosphere of 5% CO_2_/air. Viruses were isolated in C6/36 cells from serum samples collected from Myanmar by using a previously published protocol [31].

### Cell culture ELISA

Monolayers of cells in 96-well plates (Nunc) were infected with 200 µl tenfold dilution of DENV and incubated for 8 days. Culture supernatant from each culture was removed and cell monolayers were fixed with 5% (v/v) formaldehyde in PBS for 30 minutes at room temperature. After four washes with PBS-T, 100 µl 0.5% (v/v) triton X-100 in PBS was added to the monolayers for 5 minutes at room temperature. After a further three washes with PBS-T, the cells were blocked with 2% (w/v) skim milk in PBS for 30 minutes at room temperature. HRP labelled 6B6C-1 antibody [32] was diluted 1∶3000 with 2% (w/v) skim milk in PBS and 100 µl were added to each well and incubated for 1 hour at room temperature. Cells then were washed six times with PBS-T and 50 µl TMB solution was added. Virus infected cells were stained blue.

### Immunofluorescence assays

Confluent adherent (C6/36, BHK-21 clone 15, HuH-7 and HS1) and non-adherent (K562 and U937) cells in 12 well plates were infected with 200 µl DENV (DENV-1, -2, -3, -4) in serum-free RPMI-1640. After 1 hour of infection, 1 ml RPMI-1640 containing 2% v/v FBS was added to each well and incubated for 12 days. Supernatant from each culture was removed after 12 days. 5 µl of cell suspensions from each dilution were added to each well of a 12 well, teflon coated, immunofluorescence slides (ICN Biomedicals). Excess liquid was aspirated from the spots and cells were air dried for 15 minutes at room temperature before being fixed in ice cold acetone for 4 minutes. 50 µl of a anti-DENV monoclonal antibody solution (M10 for anti- DENV-1, 3H5 for anti-DENV-2, 5D4 for anti-DENV-3 and 1H10 for anti-DENV-4) [33] were added to each spot and incubated for one hour at room temperature. The slides then were washed three times with PBS, each for 10 minutes. 50 µl of a secondary antibody solution composed of a 1∶30 dilution of fluorescein isothiocyanate (FITC) labeled anti-mouse IgG (Dako) and a 1∶80 dilution of FITC labeled rabbit anti-human IgM (Dako) in PBS was added to each spot and incubated for 45 minutes at room temperature. The slides were washed with PBS three times again each for 10 minutes. Cover slips were mounted on the slides and the cells examined under a fluorescent microscope (Eclipse, Nikon) using ploem illumination. The images were recorded using photometric CoolSnap (Nikon). Cells were considered infected if a clear green fluorescence was observed in the cytoplasm of the infected cells. Fluorescent and transmitted light images were recorded for each field.

### Indirect ELISA

50 µl of supernatant from DENV infected, or uninfected cell cultures were diluted with an equal volume of chilled borate saline (B.S) pH 9.0 (B.S) and added to 96 well ELISA plates (Maxisorb, Nunc) at 4°C for 24 hours. The plates then were washed five times with PBS-T and 100 µl of HRP-labeled 6B6C-1 diluted in 1∶6000 in PBS-T was added to each well and incubated for 45 minutes at room temperature. The plates then were washed six times with PBS-T and 100 µl soluble TMB (ELISA Systems) was added to each well and incubated for 20 minutes for colour to develop. 50 µl of 1 M sulphuric acid (H_2_SO_4_) was added to each well to stop the reaction. The absorbance in each well was determined with an ELISA plate reader (Beckman Instruments) at a wavelength of 450 nm against a blank of 620 nm.

### Assay for distribution of fitness

DENVs were diluted two-fold (1 in 2 to 1 in 128) to provide a theoretical “one infectious unit” of virus as an input to individual cell cultures in 96-well plates i.e. ∼66 of the 96 well C6/36 cells monolayer’s were infected. Two wells in each 96-well plate (A1 and B1) were infected with undiluted DENV as control (Fig. S1). Yield of prototypes strains of DENV, in cultures of *A. albopictus* (C6/36) cells, infected with ten-fold dilutions was peak at about 8 days irrespective of MOI (Figure S2). Eight days after infection, the culture supernatant from each of the 96-wells was transferred into corresponding wells in a second 96-well plate. The amount of virus released from cells in each culture was determined by indirect ELISA in the second plate described previously (Fig. S1). We calculated mean and +/−2 standard deviations of the control values to determined 95 confidence interval of the range for statistically valid comparison with the fitness of individual populations. Cell monolayers in the original plate were stained for DENV E protein by cell ELISA as described previously.

### RNA extraction and RT-PCR and sequencing

RNA was extracted from 140 µl samples of virus using the QIAamp Viral RNA mini kit (Qiagen), according to the manufacturer’s instructions. RNA was quantified by spectrophotometry. Equal amounts of RNA were used for RT. Complementary DNA (cDNA) was produced from the RNA of DENV using random hexanucleotide primers (Boehringer Mannheim) and expand reverse transcriptase (Expand RT; Roche). Briefly, 1 µl random hexamer primers (200 ng/µl) was added to 11 µl RNA in a 0.5 ml tube (LabAdvantage) and the mixture was incubated at 65°C for 5 minutes in a heating block before being placed on ice for 2 minutes. Four microliters of 5x RT buffer (Roche), 1 µl 100 mM DTT (Roche), 1 µl 10 mM dNTPs (Roche), 1 µl RNAse inhibitor (40 unit/µl; Roche) and 1 µl expand RT (50 unit/µl) were added to the tube and the volume made up to 20 µl with nuclease free water. RT reactions were incubated at 55°C for 1.5 hours. The primers used for PCR amplification corresponded to a region of the E of DENV-1, which were: D1 843F, 5′-ATGCCATAGGAACATCC 3′ and D1 2465R, 5′-TTGGTGACAAAAATGCC 3′. Five microliters of 10x Expand high fidelity PCR buffer with 15 mM MgCl2 (Roche), 1 µl 10 mM dNTP, 2 µl forward primer (100 ng/µl), 2 µl reverse primer (100 ng/µl), 0.75 µl Expand high fidelity PCR system (3.5 unit/µl; Roche), 5 µl cDNA and 34.25 µl nuclease free water were mixed to make the total volume of 50 µl. PCR was performed using cycling conditions of 94°C for 2 minutes for one cycle and then 92°C for 30 seconds, 58°C for 40 seconds and 68°C for 2.30 minutes for 10 cycles, 92°C for 30 seconds, 58°C for 30 seconds and 68°C for 3 minutes for 10 cycles, 92°C for 30 seconds, 58°C for 30 seconds and 68°C for 3.30 minutes for 18 cycles run for 39 cycles followed by 68°C for 10 minutes for final extension. PCR products were electrophoresed on 1.0% agarose in 1x TBE buffer and products of the correct size were gel purified with the MinElute PCR purification kit (Qiagen), according to the manufacturer’s instructions. The purified DNA (100 ng per 300 bp of product) was added to 3.2 pmol of oligonucleotide primers (forward and reverse) in a final volume of 12 µL. The remaining sequencing reaction was performed by Australian Genome Research Facility Ltd (AGRF), Brisbane. Sequencing was performed on automated ABI 3730 DNA Analyzer (Applied Biosystems) using dye-terminator chemistry.

### Sequence Alignments and Phylogenetic Analysis

Alignment of the consensus sequences were performed using the ClustalW program in the Geneious Pro 6.1. The aligned nucleic acid sequences were used to construct bootstrapping phylogenetic tree using the Neighbor-joing tree building method and Tamura-Nei genetic distance model in the Geneious Pro 6.1.

## Results

### Phylogenetic relationship between DENV-1 isolates

**Figure 1:**
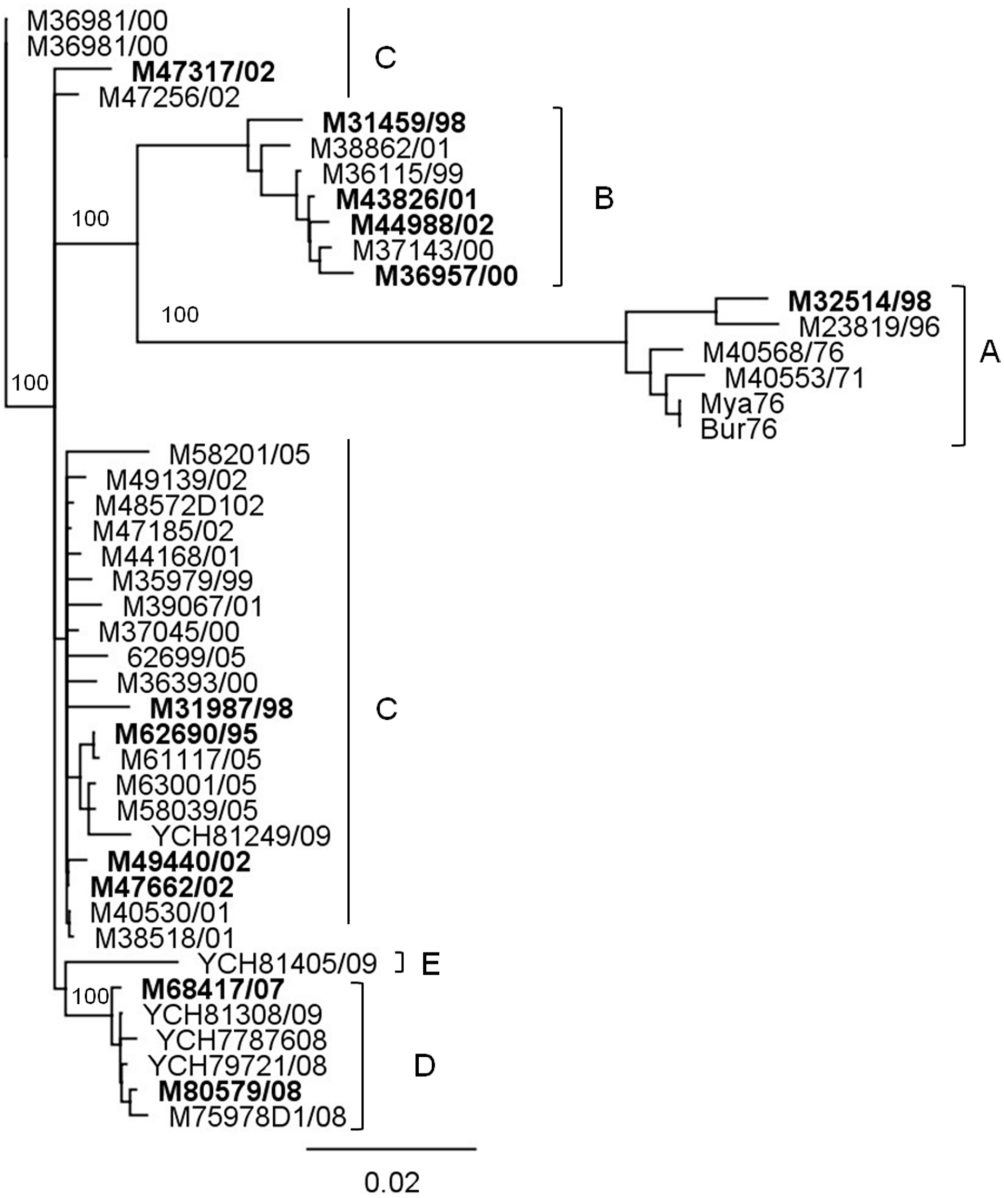
Phylogenetic analysis of the E gene of DENV-1 showing lineage extinction and replacement of DENV-1 in Myanmar. Bootstrap values (100 replications) for key nodes are shown. A distance bar is shown below the tree. Lineage A, B and E are extinct and lineage C and D are still circulating. Strains selected for study have been highlighted.

Analyses of Myanmar DENV-1 E gene sequences from Genbank and unpublished sequences (Table 1) produced a phylogenetic tree with five distinct branches (Fig. 1). Lineage A contained the first DENV-1 isolate recovered in Myanmar (Burma, Bur76 and Mya76). This lineage became extinct in 1998, about the same time lineages B and C appeared. No examples of lineage B have been recovered since 2002, but lineage C was still circulating in 2008. Lineage D was first detected in 2006, and was still circulating in 2008. The single example of lineage E, which was most closely related to DENV-1 from Vietnam, did not appear to have established cycles of transmission and was excluded from analysis. For the purposes of analysis, lineages A and B are considered to have become extinct in 1998 and 2002, respectively. Lineages C and D were deemed to be still circulating in 2008. The strains in bold type in Figure 1 were regarded as representative of their lineage and were used in subsequent studies.

### Susceptibility of vertebrate and invertebrate cell lines to infection with DENV

Because subsequent fitness studies required that DENV be cultured in both mosquito and relevant human cell lines, the susceptibility of C6/36 (mosquito) cells and a range of human cell lines (HuH7, HepG2, HC04, K562, U937, HS1 and SW987) to DENV infection was determined. Baby Hamster kidney (BHK-21) cells were included as a control substrate. With the exception of HuH7 cells, the human cell lines were uniformly refractory to infection with prototype strains of DENV (Table S1). Subsequent experiments with low passage DENV isolates and other DENV populations of interest were performed only in C6/36 and HuH7 cells (Table 2). As subsequent experiments required serum-free cell culture, the yield of DENV isolated from C6/36 cells cultured with and without a FBS supplement was assayed (Table 2) and showed no significant difference (*p*>0.05, student t-test). As previously observed (Table S1), the yield of DENV from HuH7 cells was consistently lower than that from C6/36 cells. C6/36 mosquito cells were as much as one million times more sensitive to infection with low passage strains of DENV-1. No DENV production was detectable in HuH7 cells infected with 8 of the 20 DENV strains studied.

**Table 2:** Infectivity of dengue viruses (DENVs) for mosquito (C6/36) and human (HuH7) cell lines.

Dengue virus		Titre of virus (log_10_TCID/ml)			
Serotype	Strain	C6/36 without FBS	C6/36 with FBS	HuH7 without FBS	HuH7 with FBS
DENV-1	Hawaii	6.5	5.5	<1.0	<1.0
	31459	7.0	6.5	<1.0	2.0
	31987	5.5	5.0	<1.0	3.0
	32514	7.0	6.5	3.0	3.0
	62699	6.0	4.5	<1.0	<1.0
	63001	5.5	5.0	<1.0	2.0
	75971	5.5	5.5	<1.0	2.0
	84077	8.0	7.5	3.0	3.0
	84558	7.5	7.0	<1.0	2.0
	I. C	7.5	7.0	<1.0	4.0
DENV-2	New Guinea C	8.0	8.0	4.0	4.0
	I. C	8.0	8.0	4.0	4.0
DENV-3	H87	7.5	7.5	<1.0	2.0
	82899	7.0	5.5	<1.0	<1.0
	83468	5.0	4.5	<1.0	<1.0
	84014	7.0	6.5	<1.0	<1.0
	84700	4.5	3.5	<1.0	<1.0
DENV-4	H241	7.0	7.0	<1.0	2.0
	84711	6.5	5.5	<1.0	<1.0
	84087	7.0	6.5	<1.0	<1.0

DENVs isolated from clinical patients in C6/36 were infected in C6/36 and Huh7 with ten-fold dilutions to determine the relative titres in both cell types. Both cells (C6/36 and Huh7) were infected at the same time with the same dilution of DENVs to determine the relative titres.

I.C. Infectious clone derived DENV-2.

### Distribution of fitness within population of DENV-1

**Figure 2:**
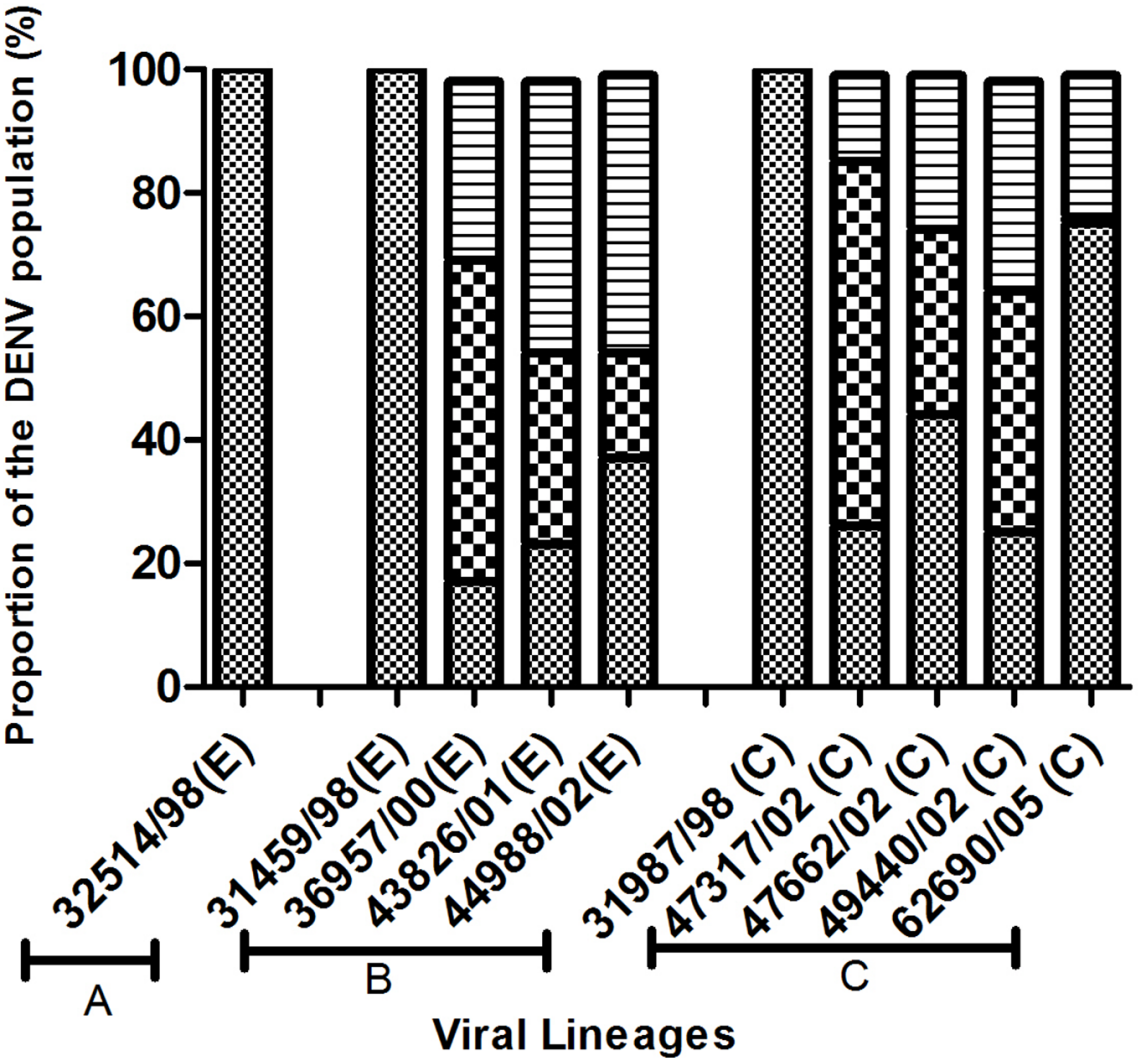
Distribution of fitness within populations of DENV-1 from four lineages. Populations are identified as strain/year/E (extinct) or C (circulating); proportion more fit than the population average indicated horizontal hatch, same fit as the original population indicated as bold squares and less fit than the original population indicated as small squares.

Stocks of viruses from the three lineages in Fig. 1 (A, B, C) were limit diluted to provide a theoretical “one infectious unit”/200 µl inoculum (i.e. 65 of the 96 wells contained infectious virus) and used to infect monolayers of C6/36 cells in 96 well plates. Eight days after infection, at the time of peak virus production (Fig. S2), the amount of virus released from C6/36 cells in each well was determined by indirect ELISA (Fig. S1). The amount of virus from cultures infected with one infectious dose of virus was compared with that of the population from which it was derived i.e. cell monolayers in the 96 well plate infected with corresponding undiluted stock virus. The mean absorbance (±2 s.d.) was calculated for the duplicate control wells containing undiluted stock of the DENV-1 population being analysed (A1, B1; Fig. S1). Supernatants from cultures giving rise to an ELISA absorbance similar to the mean (±2 s.d.) for cultures A1, B1 (undiluted stocks of virus) were regarded as having the same fitness is the population from which they were derived. Supernatants from cultures giving rise to an ELISA absorbance of more than 2 s.d. less than the mean for cultures A1 and B1 were regarded as less fit. Supernatants from cultures giving rise to an ELISA absorbance of more than 2 s.d. greater than the mean for A1 and B1 were regarded as more fit. The numerical fitness distribution within each of these classifications (more, average, less fit) is presented in Table S3. The distribution of fitness within populations of DENV-1 (lineages A, B and C) is shown in Fig. 2.

Less than 2 per cent (fewer than 1 in 94) of the members of DENV-1 populations collected in 1998 (lineages A, B, C) were more fit than the population from which they were drawn (*p*<0.05, Chi-Square-test) (Fig. 2).

Members of all populations recovered after 1998 contained some members (14.1% to 45.71%) that were more fit than the population from which they were derived except for the sample recovered in 2000 (36957/00, lineage B) which showed an increase in the prevalence of more fit members. Most of the post-1998 samples were recovered after the explosive outbreak of DENV-1 infection in Myanmar in 2001 and showed an increase in the prevalence of more fit members. While DENV populations of both lineages B and C appeared to be gaining (14.1% to 45.71%) more fit members, only lineage C has survived.

Fitness distribution in lineage B and C represents a polarization of fitness which increased in both numbers (more and less fit). We observed that 75% of the viral strains were polarized in extinct lineage B, whereas only 20% of the viral strains were polarized in circulating lineage C, (*P* = 0.09, Pearson Chi-square statistics = 2) (Table 3). We considered the fitness was being polarized when more than 33% (one third) populations were more and less fit than average fitness. We excluded linage A from this analysis because of limited number of samples for statistically valid comparison.

**Table 3:** Relationship between DENV lineage extinction and polarization in fitness in populations.

DENV fitness polarization	Lineage		Total
	Extinct	Circulating	
Polarized	3	1	4
Non-polarized	1	4	5
Total	4	5	9

75% of the extinct viral strains were polarized and 80% of the circulating strains were non-polarized. We have conducted a Chi-square test of independence to test the null hypothesis that there is no association between polarization and virus extinction. The test results show that there is no statistically significant association between polarization and virus extinction (Pearson Chi- square statistic = 2.7, *p* = 0.09).

## Discussion

DENV lineage turnover is commonly observed in DENV evolution, but it is unclear whether lineage replacement events are caused by selective pressure or by random sampling during transmission. While DENV populations are highly diverse [18], [34]–[39], the overall fitness of a DENV population in an individual host is unlikely to be simply the sum of individual fitness. Other similarly diverse arboviruses exhibit characteristics consistent with synergy within their viral populations. For example, less than 5 per cent of VSV virions within a population were more fit than the population from which they were drawn [28], and RRV populations containing less than 1% of a virulent strain nevertheless displayed a virulent phenotype [29]. Recent observations that defective DENV genomes can be complemented by fully competent genomes to overcome the defect [31] supports the concept of cooperativity within DENV populations. To date, there has been no attempt to quantify the distribution of fitness within DENV populations, and to correlate these data with epidemiological patterns. Here we report the distribution of fitness within populations of DENVs collected from Myanmar between 1998 and 2005.

Our observation that all members of DENV-1 populations collected in 1998 were less fit than the overall fitness of the populations from which they were drawn regardless of lineage (Fig. 2) is consistent with extensive complementation among the 90–99% of DENV genomes that are incapable of self-replication [16], [40], [41]. The distribution of fitness appeared to polarise in subsequent years, in which the fraction of the population that was more fit than the overall population fitness increased. This was especially apparent for lineage B from 1998 to 2002 (Fig. 2). The proportion of individuals in DENV-1 populations that were more fit than the population as a whole increased after the explosive outbreak of DENV-1 infection in 1998, suggesting that more fit viruses may have been selected during the rapid transmission accompanying the outbreak. However, an increase in the proportion of “more fit” members of a DENV population did not guarantee survival of a lineage, as lineage B became extinct between 2002 and 2005.

We observed a trend (although statistically not significant, *P* = 0.09, Pearson Chi-square statistics = 2) that fitness of extinct lineage was more polarised (>50%) than circulating lineage. It is possible that polarisation of fitness within a lineage population in which the “more fit” viruses specialising in high virus titre support the “less fit” viruses within the same population. If true, the less fit viruses presumably specialise in some other characteristics which are important for lineage survival (i.e. increased replication rate or increased ability to evade the host immune system), and similarly support more fit viruses within their lineage population. Such specialisation represents a loss of fitness homogeneity that could result in populations that are more vulnerable to stochastic sampling (bottleneck) effects. For example, the distribution of fitness of a small-sized random sample from a homogeneous 1998 lineage population would likely represent of the overall population with respect to fitness distribution, and thus would likely maintain the cooperative characteristics of the population from which it was drawn. However, the fitness distribution of a similarly small-sized random sample from the polarised 2002 lineage population would be less likely to accurately represent the fitness distribution of the source population. If the random sample were to contain insufficient numbers of more fit viruses to effectively support the less fit viruses, the lineage would become vulnerable to extinction. However, more data would be required to verify this interpretation as this is not statistically significant (*P* = 0.09, Pearson Chi-square statistics = 2) (Table 3). The results could be statistically significant if we had sufficient number of samples. Unfortunately, in this study our sample numbers are not high. Further studies required with large number of samples to determine whether fitness polarisation in DENV population has any impact on lineage extinction.

We observed no association between the distribution of fitness of members with DENV-1 populations in mosquito cells and the survival or extinction of a lineage of viruses (Fig. 2). However, there was an increase in the proportion of more fit members during and after the 2001–2002 outbreaks, suggesting that there may have been some selection for DENV that grew to high titre in mosquitoes. This observation has two caveats. The first is that there was an increase in the proportion of more fit members in population 36957/00, compared to 31459/98 (clade B) *before* the outbreak began (36957/00 was recovered in the dengue season of 2000 in which the number of reported cases was low). The second is that the half life of an *Aedes aegypti* mosquito in Thailand (and presumably in neighbouring Myanmar) is only 7–8 days [42] and so selection might be for a virus that replicated faster rather than one that grew to high titre. However, DENV, which grows to high titre, may also reach significant titres earlier.

Fitness can be defined as a measure of the ability to replicate (and produce infectious progeny) in a host [43], [44] but a more appropriate definition could be a measure of the ability to be transmitted, i.e. to infect the next host in a transmission cycle. While transmissibility is probably the most relevant measure for a virus like DENV, with infection cycles involving alternate human and mosquito hosts, technical constraints prevented this measure being used. In this study, fitness was defined as the yield of DENV-1 virions from infected cells.

An indirect ELISA procedure was employed to estimate the quantity of DENV virions released into a culture supernatant (see Materials and Methods). It was accepted that a proportion of these would contain genomes that were not infectious. However, given the complexity of the interactions between RNA genomes e.g. complementation, interference by sub-genomic RNA etc., the yield of virions was a more relevant measure of productive infection than estimates of either the number of infectious virus particles (able to infect cell substrate) or of genome copy number.

A comparison of titres of DENV in patients measured as infectious virus or as genome copy number suggested that copy number values are 10–100 times higher than infectious titres [16], [40], [41]. That the individual members of DENV populations might vary in their fitness is not surprising given that there are reports of virions with genomes with mutations and indels giving rise to intragenic stop codons as well as genomes with deletions of thousands of nucleotides [31], [34], [37], [45]–[49] There also is an extensive literature describing non-lethal changes that effect DENV replication [50]–[53]. Taken together with the comments above, it is unlikely that an individual cell is infected by a single DENV genome. For these reasons, a unit, “one infectious dose” has been used in this study and has been derived statistically, i.e. if 96 infectious units of virus in 9.6 ml are aliquoted uniformly into 96 wells of a microtitre plate, only 65 wells will contain virus (some wells will contain more than one infectious dose). While this is a weakness of this approach, there was no alternative, and the same methodology was used for all populations, so enabling comparisons to be made.

It was important to select appropriate human and mosquito cell lines for a suitable surrogate to measure fitness *in* *vitro*. The primary and/or major sites of DENV replication in humans are not known yet. Therefore, it was unclear what cells or cell lines might be appropriate substrates for experiments relevant to the human condition. A survey of the literature (Table S2) suggested DENV could be identified most commonly in the liver, as it was associated with liver dysfunction [54]–[56] and pathology [55], [57]–[59] but it was not clear whether this was due to the extensive phogocytic activity of the liver [60]–[65] or that cells in the liver are more susceptible to infection than those in other tissues. However, in this study, all human cell lines including a number of liver cell lines, were extremely refractory to infection by the low passage DENV-1 (Table 2). The use of HuH7 cells in this study reflected that these cells appeared to be the best available rather than that they were a productive cell substrate. Other investigators [66] have struggled to find human cell lines that are uniformly susceptible to infection by DENV from patient serum or by low passage DENV isolates.

These investigations focussed on DENV infections in C6/36 mosquito cells. Mosquito cell is not a perfect representation to measure fitness; however, it is representation of mosquito vectors. Additional information may have been revealed if similar studies were undertaken in human cells, and more informative changes may have been revealed if similar studies were undertaken in human cells. However, it was not possible to identify a human cell line that was sufficiently susceptible to infection with all low passage strains of DENV (Table 2). Furthermore, the most susceptible human cell line, HuH7, required a FBS supplement for growth and the FBS reduced the sensitivity of the ELISA method employed to quantitate DENV in culture supernatants.

This study has provided clear evidence that the lineage turnover in DENV transmission is not due to any selective pressures because of the variation in fitness within populations. While we observed a trend that fitness of extinct lineage DENV populations was more polarised than circulating lineage, impact of polarisation of fitness in population in DENV lineage extinction need to be further explored. As Myanmar is a hyperendemic country, the presence of multiple DENV serotypes may result in complex patterns of cross-immunity, which might determine which clades survive and which become extinct [13], [30]. The explanation for clade replacement may lie with the phenotype of the host with the more susceptible hosts (host proteins able to support the replication of DENV most efficiently; the innate immune system least able to resist infection) being infected more readily after appearance of a new clade such that, after several years, the virus struggles to survive. A new clade, with a different phenotype, may be able to exploit hosts which the resident clade is struggling to infect.

## Supporting Information

Figure S1 **Distribution of fitness within populations of DENV-1.** Serial dilutions of DENV were added to 94 wells of 96 well plates containing monolayers of C6/36 cells. Undiluted virus was added to the two remaining wells shown within bracket. Eight days later the supernatants from the cultures were transferred to 96 well ELISA plates and the cell monolayers stained for DENV antigen by indirect ELISA. The amount of DENV in each supernatant was quantified by indirect ELISA.
(TIF)

Figure S2
**Yield of prototypes strains of DENV in cultures of** ***A. albopictus*** **(C6/36) cells infected with ten-fold dilutions of (a) DENV-1, (b) DENV-2, (c) DENV-3 and (d) DENV-4 (♦ 10^−1^, ▪ 10^−2^, ▴ 10^−3^, × 10^−4^, * 10^−5^, 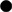 10^−6^ and + 10^−7^).**
(TIF)

Table S1 **Replication of DENV serotypes in vertebrate and invertebrate cell lines.**
(DOCX)

Table S2 **Detection of DENV in autopsy tissues of human.**
(DOCX)

Table S3 **Phenotypic diversity within population of DENV-1.** The numerical fitness distribution within each classification (more, average, less fit).
(DOCX)
