# Supplementary figures and images for: Distribution of Fitness in Populations of Dengue Viruses
Source: PLoS One. 2014 Sep 15;9(9):e107264. doi: 10.1371/journal.pone.0107264 (PMC4164612; doi:10.1371/journal.pone.0107264)

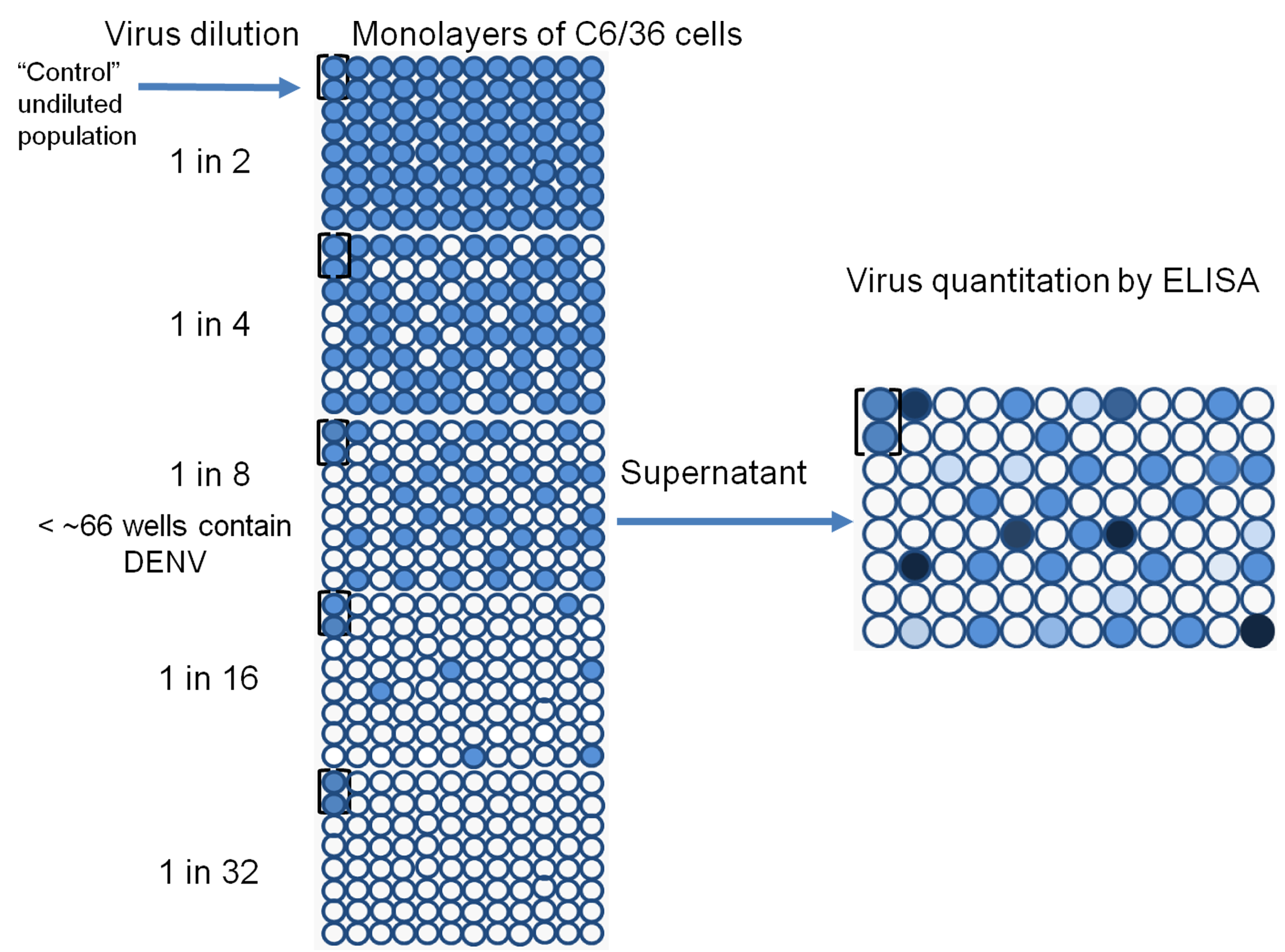

Supplement: Figure S1 — Distribution of fitness within populations of DENV-1. Serial dilutions of DENV were added to 94 wells of 96 well plates containing monolayers of C6/36 cells. Undiluted virus was added to the two remaining wells shown within bracket. Eight days later the supernatants from the cultures were transferred to 96 well ELISA plates and the cell monolayers stained for DENV antigen by indirect ELISA. The amount of DENV in each supernatant was quantified by indirect ELISA. (TIF) [file pone.0107264.s001.tif]

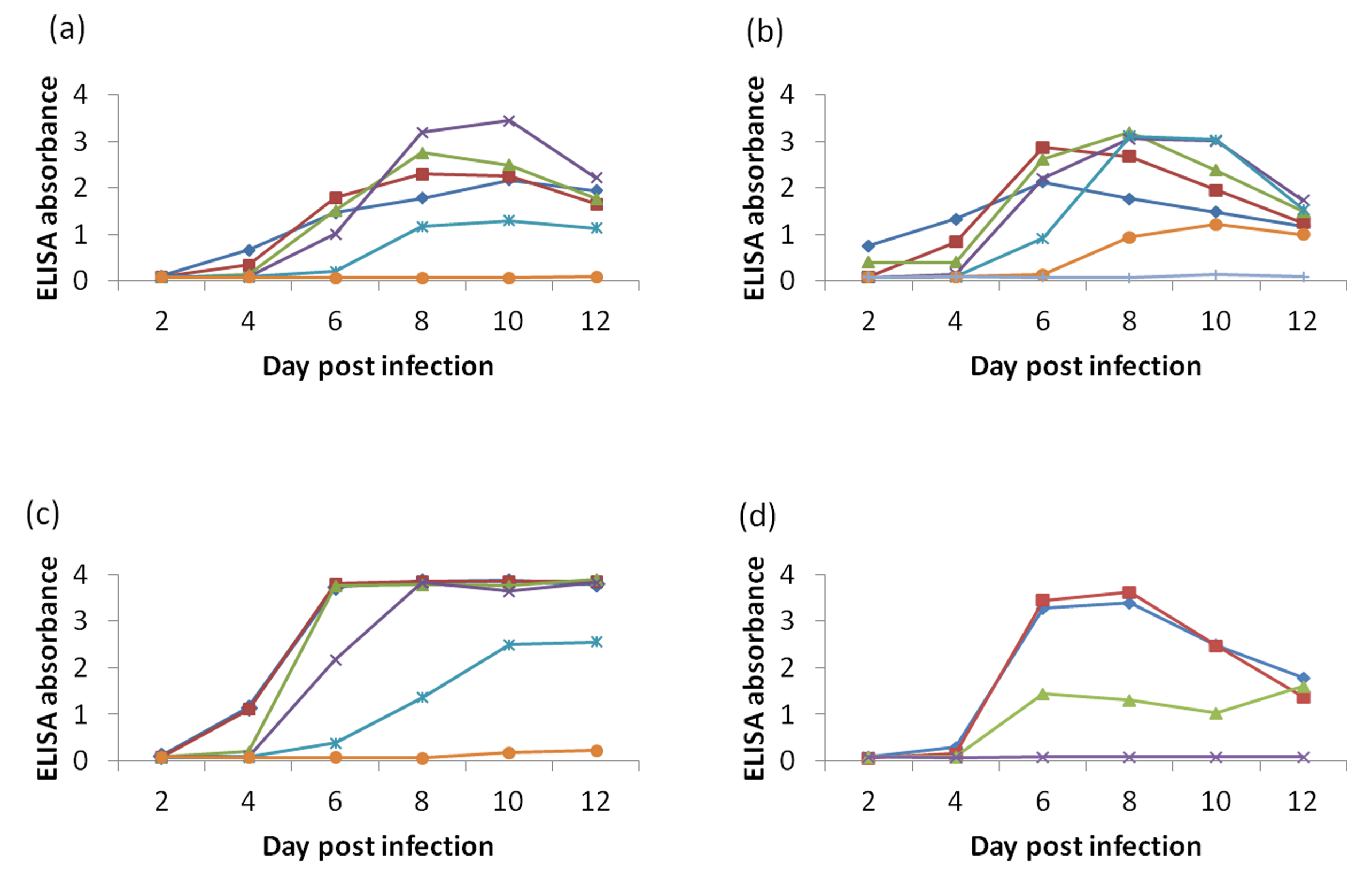

Supplement: Figure S2 — Yield of prototypes strains of DENV in cultures of A. albopictus (C6/36) cells infected with ten-fold dilutions of (a) DENV-1, (b) DENV-2, (c) DENV-3 and (d) DENV-4 (♦ 10−1, ▪ 10−2, ▴ 10−3, × 10−4, * 10−5, 10−6 and + 10−7). (TIF) [file pone.0107264.s002.tif]
