# Supplementary material for: Distribution of Fitness in Populations of Dengue Viruses
Source: PLoS One. 2014 Sep 15;9(9):e107264. doi: 10.1371/journal.pone.0107264 (PMC4164612; doi:10.1371/journal.pone.0107264)
Supplement: Table S1 — Replication of DENV serotypes in vertebrate and invertebrate cell lines. (DOCX) [file pone.0107264.s003.docx]

**Table S1. Replication of DENV serotypes in vertebrate and invertebrate cell lines.**

| **Cell line** | **Percentage of cells infected with prototype DENV serotypes at the time of peak viraemia (titres in culture supernatant [log_10_TCID50/ml])** | | | |
| --- | --- | --- | --- | --- |
|  | DENV-1 | DENV-2 | DENV-3 | DENV-4 |
| C6/36 | 57 (5.5) | 61 (6.5) | 82 (6.5) | 58 (5.5) |
| HuH7 | 0 (<1.0) | 21 (2.5) | 12 (2.5) | 10 (2.5) |
| HepG2 | n.d (<1.0) | n.d (1.0) | n.d (<1.0) | n.d (<1.0) |
| HC 04 | n.d (<1.0) | n.d (<1.0) | n.d (<1.0) | n.d (<1.0) |
| K562 | 0 (n.d) | 1 (n.d) | 0 (n.d) | 0 (n.d) |
| U937 | 0 (n.d) | 2 (n.d) | 0 (n.d) | 0 (n.d) |
| HS1 | n.d (<1.0) | n.d (4.5) | n.d (<1.0) | n.d (<1.0) |
| SW987 | n.d (<1.0) | n.d (1.0) | n.d (<1.0) | n.d (<1.0) |
| BHK-21 | 69 (3.5) | 88 (6.5) | 46 (3.5) | 41 (3.5) |
| 293T | n.d (<1.0) | n.d (<1.0) | n.d (<1.0) | n.d (<1.0) |
| COS7 | n.d (<1.0) | n.d (4.5) | n.d (1.5) | n.d (<1.0) |

n.d. not determined
