# Supplementary material for: Distribution of Fitness in Populations of Dengue Viruses
Source: PLoS One. 2014 Sep 15;9(9):e107264. doi: 10.1371/journal.pone.0107264 (PMC4164612; doi:10.1371/journal.pone.0107264)
Supplement: Table S2 — Detection of DENV in autopsy tissues of human. (DOCX) [file pone.0107264.s004.docx]

**Table S2.** **Detection of DENV in autopsy tissues of human.**

| **Authors** | **Tissues/cells involved for DENV infection** | **Techniques used for identification** | **Comments** |
| --- | --- | --- | --- |
| [1] | Liver, spleen, lung, kidney, bone marrow and blood-clot samples | Immunohistochemistry and in situ hybridization | Highest proportion of positive cells was found in spleen.  Although they were able to detect viral antigen, they were not certain about the location where viral RNA localized. |
| [2] | Liver, blood | Immunohistochemistry and RT-PCR | Clear demonstration of DENV localization in hepatocytes and kupffer cells. |
| [3] | Hepatocytes in liver | Immunohistochemistry and histopathology | Could not detect DENV antigen in spleen, lungs, heart, kidney, adrenals, intestinal tract and pancreas. |
| [4] | Liver, spleen and mesenteric lymph node | RT-PCR | Virus was only isolated from liver.  Could not detect DENV RNA in brain and kidney. |
| [5] | Brain | Immunohistochemistry and RT-PCR | Could not detect DENV antigen in other tissues. |
| [6] | Liver, macrophages in skin and lymph nodes | In situ PCR and DNA hybridization | Could not detect DENV antigen in other tissues. |
| [7] | Liver, spleen, midbrain and heart blood | Mosquito inoculation and immunoflurescence | Most DENV antigen in the liver. |
| [8] | Lymph node and bone marrow | Suckling mice and African green monkey kidney cells | Could not detect DENV antigen in liver, spleen, lungs, heart, kidney, adrenals, intestinal tract, pancreas and brain |

**References**

1. Jessie K, Fong MY, Devi S, Lam SK, Wong KT (2004) Localization of dengue virus in naturally infected human tissues, by immunohistochemistry and in situ hybridization. Journal of Infectious Diseases 189: 1411-1418.

2. Huerre MR, Lan NT, Marianneau P, Hue NB, Khun H, et al. (2001) Liver histopathology and biological correlates in five cases of fatal dengue fever in Vietnamese children. Virchows Archiv 438: 107-115.

3. Couvelard A, Marianneau P, Bedel C, Drouet MT, Vachon F, et al. (1999) Report of a fatal case of dengue infection with hepatitis: demonstration of dengue antigens in hepatocytes and liver apoptosis. Human Pathology 30: 1106-1110.

4. Rosen L, Drouet MT, Deubel V (1999) Detection of dengue virus RNA by reverse transcription-polymerase chain reaction in the liver and lymphoid organs but not in the brain in fatal human infection. American Journal of Tropical Medicine and Hygiene 61: 720-724.

5. Ramos C, Sanchez G, Pando RH, Baquera J, Hernandez D, et al. (1998) Dengue virus in the brain of a fatal case of hemorrhagic dengue fever. Journal of Neurovirology 4: 465-468.

6. Kangwanpong D, Bhamarapravati N, Lucia HL (1995) Diagnosing dengue virus infection in archived autopsy tissues by means of the in situ PCR method: a case report. Clinical and Diagnostic Virology 3: 165-172.

7. Rosen L, Khin MM, U T (1989) Recovery of virus from the liver of children with fatal dengue: reflections on the pathogenesis of the disease and its possible analogy with that of yellow fever. Research in Virology 140: 351-360.

8. Nisalak A, Halstead SB, Singharaj P, Udomsakdi S, Nye SW, et al. (1970) Observations related to pathogenesis of dengue hemorrhagic fever. 3. virologic studies of fatal disease. Yale Journal of Biology and Medicine 42: 293-310.
