# Supplementary material for: Distribution of Fitness in Populations of Dengue Viruses
Source: PLoS One. 2014 Sep 15;9(9):e107264. doi: 10.1371/journal.pone.0107264 (PMC4164612; doi:10.1371/journal.pone.0107264)
Supplement: Table S3 — Phenotypic diversity within population of DENV-1. The numerical fitness distribution within each classification (more, average, less fit). (DOCX) [file pone.0107264.s005.docx]

**Table S3.** **Phenotypic diversity within population of DENV-1. The numerical fitness distribution within each classification (more, average, less fit).**

| Strains | Extinct/ Circulating | Less fit population | Average fit population | More fit population |
| --- | --- | --- | --- | --- |
| 32514/98 | Extinct | 100 | 0 | 0 |
| 31459/98 | Extinct | 100 | 0 | 0 |
| 36957/00 | Extinct | 17.64 | 52.94 | 29.41 |
| 43826/01 | Extinct | 23.81 | 31.74 | 44.44 |
| 44988/02 | Extinct | 37.14 | 17.14 | 45.71 |
| 31987/98 | Circulating | 100 | 0 | 0 |
| 47317/02 | Circulating | 26.75 | 59.15 | 14.1 |
| 47662/02 | Circulating | 44.23 | 30.769 | 25 |
| 49440/02 | Circulating | 25.76 | 39.4 | 34.84 |
| 62690/05 | Circulating | 75 | 1.8 | 23.2 |
